# Harmonizing data on correlates of sleep in children within and across neurodevelopmental disorders: lessons learned from an Ontario Brain Institute cross-program collaboration

**DOI:** 10.3389/fninf.2024.1385526

**Published:** 2024-05-17

**Authors:** Patrick G. McPhee, Anthony L. Vaccarino, Sibel Naska, Kirk Nylen, Jose Arturo Santisteban, Rachel Chepesiuk, Andrea Andrade, Stelios Georgiades, Brendan Behan, Alana Iaboni, Flora Wan, Sabrina Aimola, Heena Cheema, Jan Willem Gorter

**Affiliations:** ^1^Department of Psychiatry and Behavioural Neurosciences, Faculty of Health Sciences, McMaster University, Hamilton, ON, Canada; ^2^Offord Centre for Child Studies, Faculty of Health Sciences, McMaster University, Hamilton, ON, Canada; ^3^School of Rehabilitation Science, Faculty of Health Sciences, McMaster University, Hamilton, ON, Canada; ^4^CanChild Centre for Childhood Disability Research, Faculty of Health Sciences, McMaster University, Hamilton, ON, Canada; ^5^Indoc Research, Toronto, ON, Canada; ^6^Ontario Brain Institute, Toronto, ON, Canada; ^7^The Centre for Addiction and Mental Health, Toronto, ON, Canada; ^8^Department of Paediatrics, Schulich School of Medicine and Dentistry, Western University, London, ON, Canada; ^9^Holland Bloorview Kids Rehabilitation Hospital, Toronto, ON, Canada; ^10^Department of Pediatrics, Faculty of Health Sciences, McMaster University, Hamilton, ON, Canada; ^11^Center of Excellence for Rehabilitation Medicine, University Medical Center Utrecht, Utrecht, Netherlands; ^12^Brain Center, University Medical Center Utrecht, Utrecht, Netherlands

**Keywords:** data, harmonization, curation, neurodevelopmental disorders, child, sleep

## Abstract

There is an increasing desire to study neurodevelopmental disorders (NDDs) together to understand commonalities to develop generic health promotion strategies and improve clinical treatment. Common data elements (CDEs) collected across studies involving children with NDDs afford an opportunity to answer clinically meaningful questions. We undertook a retrospective, secondary analysis of data pertaining to sleep in children with different NDDs collected through various research studies. The objective of this paper is to share lessons learned for data management, collation, and harmonization from a sleep study in children within and across NDDs from large, collaborative research networks in the Ontario Brain Institute (OBI). Three collaborative research networks contributed demographic data and data pertaining to sleep, internalizing symptoms, health-related quality of life, and severity of disorder for children with six different NDDs: autism spectrum disorder; attention deficit/hyperactivity disorder; obsessive compulsive disorder; intellectual disability; cerebral palsy; and epilepsy. Procedures for data harmonization, derivations, and merging were shared and examples pertaining to severity of disorder and sleep disturbances were described in detail. Important lessons emerged from data harmonizing procedures: prioritizing the collection of CDEs to ensure data completeness; ensuring unprocessed data are uploaded for harmonization in order to facilitate timely analytic procedures; the value of maintaining variable naming that is consistent with data dictionaries at time of project validation; and the value of regular meetings with the research networks to discuss and overcome challenges with data harmonization. Buy-in from all research networks involved at study inception and oversight from a centralized infrastructure (OBI) identified the importance of collaboration to collect CDEs and facilitate data harmonization to improve outcomes for children with NDDs.

## Introduction

Neurodevelopmental disorders (NDDs) are classified as a group of disorders that typically present during the developmental period and occur as a result of detriments to the developing central nervous system that affect at least one area of functioning (American Psychiatric Association, 2015). Children with NDDs may experience deficits in social, academic, personal, and/or occupational functioning, and the level of functioning may vary within each type of diagnosis (Reed et al., 2019). Although the etiologies of NDDs can vary, the development of secondary conditions or health problems occurs across conditions, suggesting that the investigation of NDDs inclusively might create equitable health solutions. To date, the majority of research in children with NDDs has focused on single diagnoses; notwithstanding value to individual NDDs, it limits the opportunity for treatment (i.e., health promotion, clinical strategies, or specific intervention) across multiple diagnoses. Rethinking the traditional medical model of treating a single disorder to that of a population approach has potential to transform healthcare (Halfon et al., 2014). Likewise, recent experimental studies have started to investigate interventions across different NDDs with similar underlying symptomology (Rigney et al., 2018; Ludyga et al., 2021). If similar (effective) treatment modalities in fact exist across diagnoses, this warrants an opportunity for a *transdiagnostic* approach to treat health issues in children with NDDs.

A common morbidity across children with NDDs is the high prevalence of sleep problems (Didden and Sigafoos, 2001). These include but are not limited to issues with sleep onset, maintenance, and early arousal from sleep (Lewandowski et al., 2011). For children with NDDs, the relationship between sleep and the condition (i.e., NDD) is often viewed as bidirectional, where the condition might contribute to poor sleep, whereas sleep problems might worsen the condition (Lewandowski et al., 2011). Most literature has focused on specific diagnoses individually and by using inconsistent assessment techniques; this results in fragmented information that is challenging to synthesize. As such, sleep problems are not uniformly defined across NDDs in children, and further insight into the associations between severity or complexity across and within conditions and sleep is warranted.

Various conditions are classified under the term NDDs (e.g., Intellectual Disability [ID], Autism Spectrum Disorder [ASD], Attention Deficit/Hyperactivity Disorder [ADHD], Obsessive Compulsive Disorder (OCD), Cerebral Palsy [CP], Epilepsy, etc.) (American Psychiatric Association, 2015). Clinical studies in these populations are often hindered by small sample sizes that are geographically isolated, and therefore limit the generalizability of the findings. Studies of larger sample sizes across NDDs that increase generalizability and external validity will improve our understanding of sleep problems and other health issues. This will enhance the ability to investigate correlates and effect modification amongst exposures and outcomes. These opportunities are pertinent to understanding sleep problems, particularly given the immediate concerns from parents of children with NDDs on their child’s development and other health impacts (Tan-MacNeill et al., 2020; Hulst et al., 2021).

The Ontario Brain Institute (OBI) was founded in 2010 with the intent to address the societal and individual costs of brain disorders across the lifespan^1^ by catalyzing discovery and innovation (Stuss, 2014; Stuss, 2015). It fosters research and clinical collaboration to promote research excellence while ensuring the perspectives of patients and families are incorporated into the research planning and process to help shape meaningful health outcomes. OBI funds and manages several research networks (herein referred to as Integrated Discovery Programs [IDPs]) that span many disciplines including NDDs, cerebral palsy, epilepsy, concussion, depression, youth mental health, and neurodegenerative disorders. The term “Integrated Discovery” describes an approach to research that is transdisciplinary that involves the collection of different types of data while being cognizant of the heterogeneity that exists within neurological disorders. The establishment of programs by symptom-based disorder allows for investigation into comorbidities that might affect various disorders differently (Stuss, 2014). Each IDP includes a diverse group of experts who follow a common protocol, methodology, ontologies, and outcome assessments (referred to as common data elements [CDEs: standardized assessments both within and across IDPs]) which facilitates the harmonization of individual participant data. However, there is an opportunity for researchers within an IDP to investigate and collect data on outcomes distinct from the common protocol that pertain to their areas of interest and expertise. Each IDP implements a common consent process, affording researchers an opportunity to work within and across data, while acknowledging the important role of patients and their caregivers participating in research. Together, this is referred to as prospective harmonization and allows for the application of a common methodology across multiple IDPs in order to perform data comparisons at point of collection (Fortier et al., 2023). To position itself as a leader in Brain Science, OBI partnered with Indoc Research^2^ and developed Brain-CODE: an advanced neuroinformatics platform with the ability to link data from provincial, national, and international institutions, and ultimately federating and analyzing large data sets to promote new discoveries to improve care for patients with brain disorders (Vaccarino et al., 2018). Brain-CODE not only supports collaborative research programs through the integration and analysis of large volumes of complex data, but also facilitates open science by making data open and accessible (Lefaivre et al., 2019; Behan et al., 2023).

The overarching objective of this paper is to describe the data management, collation, and harmonization methodologies within OBI’s cross-IDP sleep study and discuss the logistical and conceptual challenges endured and overcome in this process. The results from the cross-IDP sleep study were previously published (McPhee et al., 2023), and this manuscript speaks to the associated methodology and intricacies of data processes. This paper aims to: (1) describe the methods for collecting, organizing, and managing OBI cross-IDP data; (2) provide detailed procedures for the harmonization of CDE data; (3) discuss analytical strategies for harmonized data in the context of defining severity in NDDs; and (4) share lessons learned from data processing across research networks. We believe a transparent report of the collection, harmonization, and analytical strategies of data will provide value to other researchers interested in combining data in children with NDDs and other diagnoses.

### Collecting, organizing, and managing data

The sleep study consisted of data from the following three IDPs: CP-NET (CP); The Province of Ontario Neurodevelopmental disorders (POND) (ASD; ADHD; OCD; and ID); and EpLink (Epilepsy). Details about each IDP, including population(s), cohorts, and study designs can be found here.^3^ The sleep study team comprised investigators from each IDP, OBI research representatives, Indoc members, and a sleep expert, which was formed in October 2020 and stemmed from the idea to investigate associations between sleep disturbances, internalizing symptoms, and (HRQOL) in children with NDDs. Specifically, each IDP comprised a clinician member to provide perspective on the IDP-specific population(s) and to contextualize the research findings, a neuroinformatic lead for data support, and/or a program manager for supporting broader liaising within their respective IDP. The OBI representatives included a project manager, research manager, informatics lead, and Knowledge Translation (KT) lead; the latter to support dissemination activities. OBI supported data access to a Brain-CODE workspace and facilitated data acquisition from each IDP. The study team met biweekly to discuss planning, analysis, manuscript writing and other dissemination activities (e.g., webinars for children and families with NDDs).

**Figure 1:**
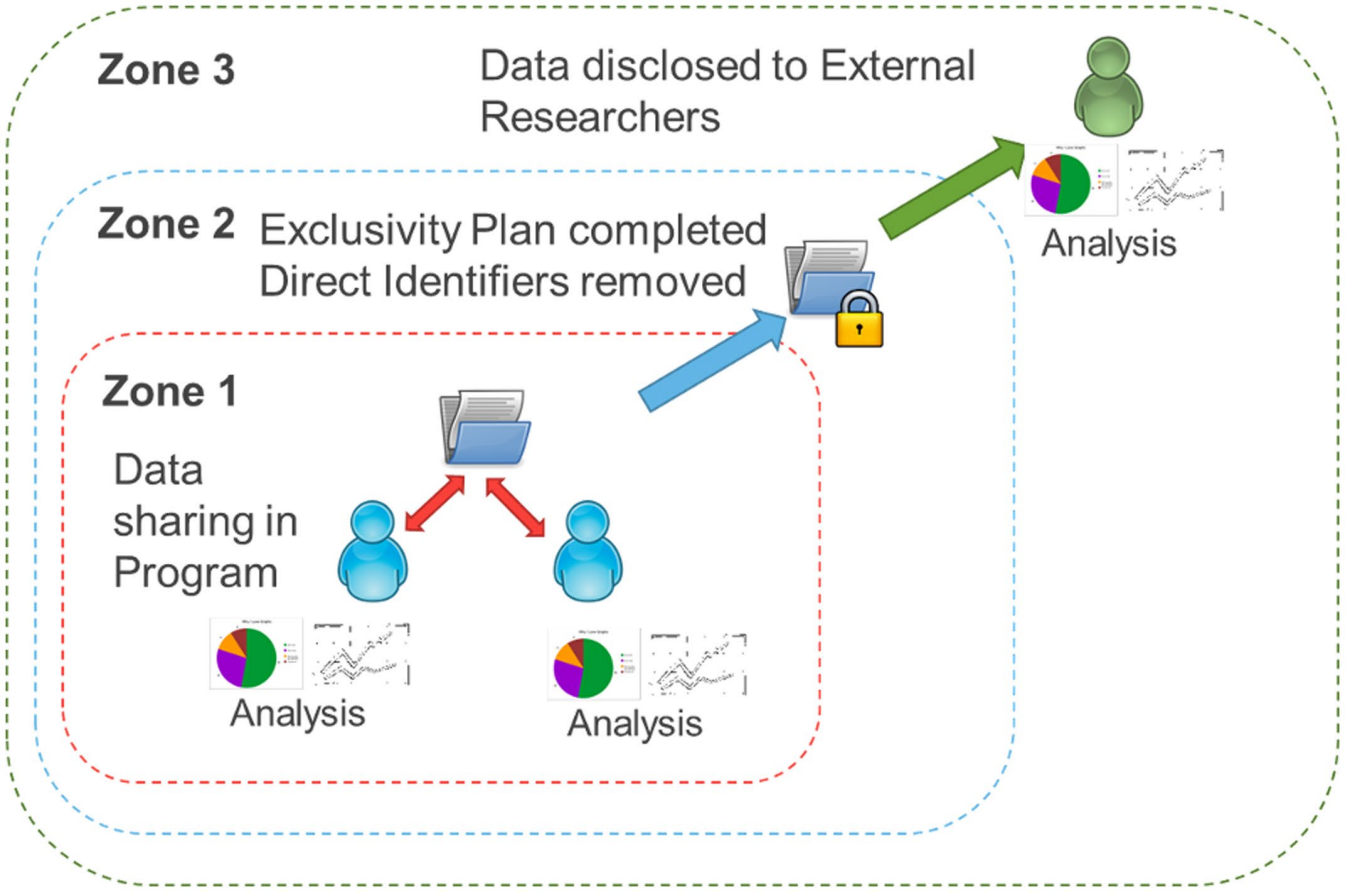
Brain-CODE’s structured format of data. Zone 1: raw data collected through Integrated Discovery Programs is stored in the database. Zone 2: personal identifiers are removed and data securely linked with external databases. Zone 3: external researchers can request access to the data.

OBI has developed and implemented a Brain-CODE Participation Agreement and a standardized Data Use Agreement (DUA). The Participation Agreement with each IDP facilitates upload and sharing within that respective IDP, while the DUA facilitates the sharing of data for secondary purposes (Lefaivre et al., 2019). Prior to initiating data collection, each IDP study undergoes rigorous project validation to ensure that variable naming, field naming, item coding, case report forms, data dictionary and data exports are in compliance and consistent with Brain-CODE standards (Vaccarino et al., 2018). Essentially, Brain-CODE ensures adherence to data standards and the equivalence across IDPs to facilitate data harmonization, such that data formatting from one IDP is consistent with data from another. Brain-CODE provides a virtual laboratory environment where IDPs or data producers can upload, download, manage, curate, and share their data with study collaborators. OBI developed and implemented in Brain-CODE a highly secure, three-zone infrastructure for controlled access of data. Briefly, Zone 1 supports the electronic capture and transfer of raw data provided to OBI by data producers affiliated with participating institutions where only direct study collaborators can access the data; Zone 2 is a virtual space containing data that have been processed to remove direct identifiers; and Zone 3 is a virtual workspace where data are disclosed to external researchers in alignment with their data access request (Figure 1); (Lefaivre et al., 2019). For the sleep study (McPhee et al., 2023), the project required access to Zone 1 data. A research ethics board (REB) application was submitted (McMaster University; Hamilton integrated Research Ethics Board [HiREB]) for approval to access and analyze previously collected data. The REB application was for a retrospective analysis, and contained a detailed proposal and statistical analysis plan for the secondary use of data to understand the prevalence of sleep disturbances and associations between sleep, internalizing symptoms (anxiety and depression), and health-related quality of life in children with NDDs. Upon receiving ethics approval (HiREB #12801), the first author (PGM), under the supervision of the senior author (JWG) (both CP-NET Investigators) worked with OBI and the IDPs to execute a Zone 1 DUA to permit sharing of data across programs.

OBI adopted a set of standardized assessments to facilitate the collection and sharing of participant level data, referred to as CDEs. These CDEs include both demographic and clinical variables and were decided upon by participating investigators in each IDP following a rigorous consensus procedure (i.e., Delphi process) (Vaccarino et al., 2018, 2022). A comprehensive list of the Brain-CODE CDEs can be found on the Brain-CODE website.^4^ Demographic variables in the sleep study included sex, date of birth (to derive age), ethnicity, and primary caregiver’s socioeconomic status (including income and level of education). Clinical variables pertained to mental health and behaviors, and included HRQOL, as well as clinical endpoints of internalizing symptoms and sleep (McPhee et al., 2023). A detailed description of the CDEs included in this study is presented in Table 1.

**Table 1:** Common data elements in the cross-integrated discovery program sleep study.

CDE	Question(s)	Response option(s)
Brain-CODE demographic form	What is the subject’s date of birth?	DD/MMM/YYYY
Brain-CODE demographic form	What is the sex of the subject?	Female Male Unknown Unspecified
Brain-CODE demographic form	What is the ethnicity of the subject?	Aboriginal Arab Black Chinese East Asian Filipino Japanese Jewish Korean Latin American/Hispanic South Asian Southeast Asian West Asian White Other ethnic group Do not know Prefer not to answer
Brain-CODE demographic form	What is the subject’s primary caregiver’s highest grade or level of school completed or the highest degree obtained?	Never attended/Kindergarten only 1st grade 2nd grade 3rd grade 4th grade 5th grade 6th grade 7th grade 8th grade 9th grade 10th grade 11th grade 12th grade, no diploma High school graduate GED or equivalent Some college, no degree Associate degree: occupational/technical/vocational program Associate degree: academic program Bachelor’s degree (e.g.; BA; AB; BS; BBA) Master’s degree (e.g.; MA; MS; MEng; MEd; MBA) Professional school degree (e.g.; MD; DDS; DVM; JD) Doctoral degree (e.g.; PhD; EdD) Unknown
Brain-CODE demographic form	What is the subject’s approximate total household income (from all sources) before taxes last year?	Less than $10,000 $10,000–$24,999 $25,000–$49,999 $50,000–$74,999 $75,000–$99,999 $100,000–$149,999 $150,000–$199,999 $200,000 or more Do not know Prefer not to answer
Child’s sleep habits questionnaire (CSHQ)	45 questions pertaining to sleep habits and possible difficulties with sleep	Usually (5 or more times/week) Sometimes (2–4 times/week) Rarely (never or 1 time/week)
KINDL-R	24 questions pertaining to child’s well-being and health-related quality of life	Never Seldom Sometimes Often All the time
Revised children’s anxiety and depression scale (RCADS)	47 questions pertaining to internalizing symptoms (anxiety and depression) of the child	Never Sometimes Often Always

Upon receiving ethics approval (HiREB #12801) (December 2020), DUAs were signed and approved (DUAs were signed by OBI, the three contributing IDPs, and lead investigators using the data [PGM and JWG]), and the first author (PGM) was provided access to a virtual workspace via Virtual Private Network (VPN) and remote desktop applications to review and download data through LabKey.^5^ Importantly, analytics are brought to the data to minimize data transfer load and reduce data risks; each IDP uploaded and managed data as .csv files in Brain-CODE using LabKey platform. The virtual workspace was customized and equipped with STATA statistical software package (version 16.1) to generate syntax and execute the statistical analysis plan for the sleep study (McPhee et al., 2023).

### Data harmonization

The cross-IDP sleep study accessed and utilized Zone 1 data, which can contain personal health information that has been approved for upload by institutional research ethics boards. Data in Zone 1 are coded (i.e., de-identified) by replacing identifying information with a unique subject identifier. Subject identifiers adhere to a standardized naming convention that is consistent across all datasets in Brain-CODE to inform a common understanding (Lefaivre et al., 2019). Data pertaining to the relevant CDEs for the sleep study and containing subject identifiers (i.e., de-identified data) were processed in LabKey.

To begin, data files were converted from .csv format to .dta and uploaded into STATA statistical software program. The first author cross-referenced data from each IDP with data dictionaries to inform harmonization. Renaming, sorting, and replacing variable names were performed at this stage to ensure consistency across IDPs, and to facilitate statistical analyses. Key considerations for each CDE and data presentation were checked for the following: consistent/inconsistent labeling; qualitative vs. numeric responses; checks for missingness; and assessments for outliers and anomalies. Any inconsistencies or obscurities were clarified through individual meetings between the first author and neuroinformatic leads (i.e., an individual within an IDP responsible for overseeing and managing informatics related activities) from each IDP. Harmonized variables were created initially within each IDP study master file and subsequently combined (i.e., merged) to create a single data file for analysis. Demographic variables were straightforward (Table 1): age was derived as time in years between date of birth and baseline assessment; there were 15 response options for ethnicity – nearly 75% of participants were Caucasian. As such, ethnicity was dichotomized into Caucasian or Other; there were 22 response options for primary caregiver’s highest level of education – for analytic purposes, we derived education into the following categories: less than high school, high school diploma, university or college degree, master’s degree, and doctoral or professional degree; gross household income had eight response options – for analytic purposes, we derived income into the following categories: <$50,000, $50,000–$99,999, $100,000–$199,999, and >$200,000. An operationalization of “severity” of each NDD was derived through multiple discussions with the study team. Specific details pertaining to this process are described below. Sleep disturbances, internalizing symptoms, and HRQOL were assessed using the Children’s Sleep Habits Questionnaire (CSHQ; Owens et al., 2000), Revised Children’s Anxiety and Depression Scale (RCADS; Chorpita et al., 2000), and KINDL-R (Ravens-Sieberer and Bullinger, 1998), respectively. Subscales and total scoring procedures for the CSHQ, RCADS, and KINDL-R followed each instrument’s rating instructions. Specific details for the CSHQ are described below. Unfortunately, we did not have information for all participants pertaining to medications, concomitant disorders, or any diagnostic overlap.

### Examples of analytical strategies for harmonized data

#### Severity of disorder

The study team was interested in understanding the associations between severity of disorder and sleep disturbances and HRQOL in children with NDDs. However, there was not a CDE that captured severity of disorder or daily functional limitations. The study team met and discussed how severity of disorder could be qualified for this study. Each IDP proposed a method for defining severity of disorder based on research literature and clinical expertise. For CP, severity of disorder was defined using the Gross Motor Function Classification System (GMFCS; Palisano et al., 2008). Participants were defined as community ambulatory (less severe = GMFCS levels I and II) or community non-ambulatory (more severe = GMFCS levels III, IV, and V; McPhee et al., 2015). Severity of ASD was defined using a clinical threshold in the Social Communications Questionnaire (SCQ; Eaves et al., 2006): a score < 15 was defined as less severe; a score ≥ 15 was defined as more severe. Severity of ADHD was defined using evaluations from the Strengths and Weaknesses of ADHD-symptoms and Normal-behavior (SWAN) rating scale (Swanson et al., 2012): a total score < 5 points in inattentive or hyperactive impulsive subscales was defined as less severe; a total ≥ 6 points was defined as more severe. Severity of OCD was defined using the Toronto-Obsessive-Compulsive Scale (>0 = more severe; Park et al., 2016). Epilepsy severity was defined by use of anti-epileptic medication (Kwan et al., 2010): <2 anti-epileptic drugs was defined as less severe; ≥2 anti-epileptic drugs was defined as more severe for the purpose of this study. Unfortunately, there was no assessment of intelligence quotient and children with ID were not classified based on severity; this is acknowledged as a limitation in our study (McPhee et al., 2023). After dichotomizing severity for each disorder (with the exception of ID), severity was harmonized by each disorder and totalled across disorders (1 = less severe; 2 = more severe). Chi-squared tests were performed to understand the relationship between severity of disorder and rates of pediatric sleep disorders. Severity of disorder was included as a dichotomous covariate to understand the association between severity of disorder and HRQOL. For the 1,438 children with NDDs included in this study, severity of disorder was defined in 1,367 (95.1%).

#### Children’s sleep habits questionnaire

The CSHQ was developed to assess common sleep problems in children. This 45-item outcome measure evaluates the child’s sleep based on behaviors pertaining to eight different subscales: bedtime resistance (6 items), sleep onset delay (1 item), sleep duration (3 items), sleep anxiety (4 items), night wakings (3 items), parasomnias (7 items), sleep disordered breathing (3 items), and daytime sleepiness (8 items). Response options pertain to frequency and range from usually (1) to rarely (3). Thirty-three items are totaled to generate a Total Sleep Disturbance Index (TSDI), with a score ≥ 41 indicative of a pediatric sleep disorder (Owens et al., 2000). Missing values are prorated by using the average score from the following subscales provided no more than two items are missing: bedtime resistance, sleep anxiety, parasomnias, and daytime sleepiness. Data harmonization for each IDP for the CSHQ discovered that an abbreviated version of the CSHQ (CSHQ-A – 22 items; SECCYD-Wisconsin N, 2017) was administered and collected at baseline in one of two studies from CP-NET. The decision to use the CSHQ-A instead of the CSHQ was made to reduce participant burden. Only two (sleep onset delay and night wakings) of the eight subscales could be derived using the CSHQ-A, and TSDI was not derived for those completing the CSHQ-A. Therefore, we observed subscale and TSDI scores as follows: bedtime resistance (*n* = 916 [63.7%]), sleep onset delay (*n* = 1,230 [85.5%]), sleep duration (*n* = 992 [69.0%]), sleep anxiety (*n* = 955 [66.4%]), night wakings (*n* = 1,168 [81.2%]), parasomnias (*n* = 930 [64.7%]), sleep disordered breathing (*n* = 950 [66.1%]), daytime sleepiness (*n* = 961 [66.8%]), and TSDI (*n* = 838 [58.3%]; McPhee et al., 2023).

## Discussion

Given the heterogeneous nature of many brain-based disorders, including NDDs, and the overlap between diagnoses in clinical populations, it is important to understand commonalities in pathophysiologies and behaviors of these populations. This paper provides detailed information on the harmonization, analytical derivations, and strategies developed to examine demographic, mental health, and behavioral (e.g., sleep) data across different research programs encompassing children with NDDs. OBI is a leader in Brain Science and the cross-IDP harmonization described herein is a foundation for future data linkages to address clinical research gaps and ultimately improve care for individuals with brain-based disorders. As a foundation for OBI, its current and future collaborators, and researchers embarking on similar data harmonization, this transparent methodological- and lessons learned-style paper is a reference for future research. This section will reflect on the challenges and successes of the project, while discussing lessons learned and future opportunities for data harmonization in brain-based disorders research.

### Lesson: prioritize common data elements

OBI and its neuroinformatics platform (Brain-CODE) are positioned to facilitate data linkages from provincial, national, and international databases. At this time, six provincial IDPs funded by OBI utilized the CDEs and contributed data to Brain-CODE, and barriers and facilitators to understanding data collection and collation strategies within each IDP were common.

A main barrier to data harmonization across IDPs is the prioritization of CDEs in individual study designs. Principal Investigators tend to be invested in their respective research domain and to pivot to be collaborative and including CDEs does require investment and support for change management. Despite implementing a consensus procedure (i.e., Delphi process) to derive CDEs to be collected by each IDP, which included the consideration of feasibility concerns (e.g., participant and clinician burden), there remained variability in the consistency of CDE collection within and across research programs. This was discovered *post hoc* in a team study meeting for the cross-IDP project; an IDP perceived the CSHQ to be a time burden for participants, and implemented an abbreviated CSHQ instead. Indeed, participant burden in research is common and often relates to excessive time required to complete a battery of outcome measures (Rolstad et al., 2011). Despite this approach to mitigate participant burden, the opportunity to harmonize data from an abbreviated measure with the complete CSHQ was limited. As a result, a substantial number of participants did not have TSDI derived, and were removed listwise from analysis for this study outcome. Ultimately, this discovery by the steering committee on the cross-IDP sleep study resulted in extra time and effort to understand and overcome this data harmonization barrier. Maintaining effective and timely communication through biweekly study meetings for the cross-IDP project identified this issue and facilitated discussion and decisions to best carry forward the analysis. The decision to implement an abbreviated outcome measure should first be brought to OBI and, if approved, included in the data dictionary for the IDP.

### Lesson: ensure consistent variable naming and a detailed data dictionary

OBI is positioned to facilitate data sharing in an efficient and highly secure infrastructure. OBI developed a Brain-CODE Participation Agreement that is completed by all IDPs and institutions prior to transferring data to Brain-CODE. Brain-CODE’s “privacy by design” approach facilitates the highest level of data utility while protecting study participant privacy (Lefaivre et al., 2019). Data collection at the individual participant level is overseen by lead investigators within each IDP, after which data are uploaded to Brain-CODE. This approach is ideal as it combines analytical opportunities and compliance with ethical requirements and participant confidentiality (Lefaivre et al., 2019). However, ensuring that data dictionaries and variable naming are consistent from study design inception through to data harmonization and analysis is instrumental. At the beginning of study design, project validation is performed by Brain-CODE to ensure variable naming, field naming, item coding, case report forms, data dictionary and data exports are consistent across IDPs (Vaccarino et al., 2018). Ideally, this standardization and adherence to data dictionaries implemented at the start of data collection should facilitate methodological clarity. However, there were instances in this project where inconsistent variable naming, incorrect variable coding, and premature derivations proved to be time consuming and burdensome on the cross-IDP sleep study steering committee. For instance, primary caregiver’s education level was reverse scored in two studies from one IDP. Fortunately, this was identified since columns for qualitative and numeric responses for education were available, and careful observation and correction from the analytic team was performed to overcome this error. Likewise, subtle differences in variable naming across IDPs were captured during data merging attempts in STATA statistical software program. It is imperative that variable naming is identical to ensure facilitation of data merging (Kohler and Kreuter, 2005). Name changes to adhere to within-IDP-study data dictionaries were implemented by the study team to allow for merging. Finally, in an attempt to prepare for data derivations, some data were reverse scored prior to uploading into Brain-CODE and LabKey. Fortunately, reverse scored items were identified by unique variable names, which ensured that the first author did not re-reverse score the data. Future uploads of CDE data should remain unprocessed and allow the analytic team the control and ability to implement proper derivation and scoring of items and outcome measures to promote prospective harmonization. The opportunity for automatically scoring an outcome measurement instrument, if desired, should be agreed upon, tested, and performed where possible (Ercole et al., 2020). An example of a CDE that can be scored automatically using syntax available from the developer is the RCADS (Chorpita et al., 2000).

### Future directions

To date, the cross-IDP sleep study described herein is one of two initial cross-program analyses within OBI (Vaccarino et al., 2022; McPhee et al., 2023). The process itself commenced with the study idea in October 2020, with REB approval in December 2020 and study completion and manuscript submission in less than 1 year (November 2021). The utility and collection of CDEs across IDPs allows OBI to ask and answer clinical questions that address gaps in the literature and that are meaningful to patients and families with brain-based disorders. Importantly, OBI is invested in expanding its collaborations beyond the provincial level, and is seeking collaborations from other institutions and organizations that share common interests in brain disorders. Indeed, OBI’s Brain-CODE architecture was recently adopted and implemented in clinical and research settings at the Centre for Addiction and Mental Health (Rotenberg et al., 2018). With much research to date in brain disorders, particularly children with NDDs, being limited to studies of single disorders and of small sample sizes, the external validity or generalizability is often limited. OBI is positioned to make new discoveries at population levels that will inform screening, management, and treatment for better care for individuals with brain-based disorders. Future consideration of other collaborators’ needs to underscore the importance of prioritizing CDEs, while being cognizant of the transparency and consistency of data collection, federation, and harmonization and work required at both time of data collection and analysis phases are warranted. Consistent with these needs, an internal recommendation from OBI is for an even distribution of work between IDPs and end-users, such that data are organized in a format consistent with data dictionaries prior to uploading to LabKey for external use; doing so will promote prospective rather than retrospective data harmonization and analysis. Additionally, the implementation and utilization of data quality and data integration tools that follow a framework for data curation and harmonization and promote automation and improve validity and transformation of data should be considered. Lastly, another relevant opportunity is to implement classical test theory practices to validate CDEs both within and across brain-based disorders.

### Limitations

Although a strength of this paper is the transparent descriptions of data harmonization and analytical procedures across different IDPs, important limitations should be noted. Despite the implementation of CDEs, common naming procedures, and data dictionaries across IDPs, we were unable to capture or include any verbal instructions given to participants by research staff at time of data collection; thus, response bias should be cautioned. A strength of OBI is its position as a large brain disorder research network; however, not all brain-based disorders or NDDs are represented. This emphasizes a need for future collaborations with other organizations to increase the representation of different disorders.

## Conclusion

Overcoming the traditional approach of researching in “silos” affords an opportunity to understand similarities and differences between children with NDDs, which could support common interventions for health promotion, surveillance, and management. Different challenges of collecting, harmonizing, and analyzing data across different disorders have been identified and suggestions to overcome them will further our understanding of health outcomes in children with NDDs: these include the utilization of data quality tools and data integration tools that promote automation and that can be helpful to improve validity and transformation of data throughout data curation and promote prospective data harmonization. Buy-in from IDPs at study inception and oversight from OBI’s infrastructure identify the importance of collaboration to improve the generalizability of findings in this population.

## Data availability statement

The data analyzed in this study is subject to the following licenses/restrictions: The original contributions included in this article are previously published; further inquiries can be made to the corresponding author (PGM). Requests to access these datasets should be directed to info@braininstitute.ca.

## Ethics statement

The studies involving humans were approved by Hamilton Integrated Research Ethics Board (REB #12801). The studies were conducted in accordance with the local legislation and institutional requirements. Written informed consent for participation in this study was provided by the participants’ legal guardians/next of kin.

## Author contributions

PGM: Conceptualization, Data curation, Formal analysis, Methodology, Validation, Writing – original draft, Writing – review & editing. ALV: Conceptualization, Investigation, Methodology, Validation, Writing – original draft, Writing – review & editing. SN: Conceptualization, Methodology, Writing – original draft, Writing – review & editing. KN: Conceptualization, Writing – original draft, Writing – review & editing. JS: Conceptualization, Writing – original draft, Writing – review & editing. RC: Conceptualization, Writing – original draft, Writing – review & editing. AA: Conceptualization, Writing – original draft, Writing – review & editing. SG: Conceptualization, Writing – original draft, Writing – review & editing. BB: Conceptualization, Methodology, Writing – original draft, Writing – review & editing. AI: Conceptualization, Writing – original draft, Writing – review & editing. FW: Conceptualization, Writing – original draft, Writing – review & editing. SA: Conceptualization, Writing – original draft, Writing – review & editing. HC: Conceptualization, Methodology, Writing – original draft, Writing – review & editing. JWG: Conceptualization, Investigation, Methodology, Supervision, Validation, Writing – original draft, Writing – review & editing.

## Footnotes

^1^ https://braininstitute.ca

^2^ https://www.indocresearch.org/index.html

^3^ https://braininstitute.ca/research-data-sharing

^4^ https://www.braincode.ca/content/getting-started#toc-2

^5^ https://www.labkey.com
